# Unified Sampling and Ranking for Protein Docking with DFMDock

**DOI:** 10.1101/2024.09.27.615401

**Published:** 2024-09-28

**Authors:** Lee-Shin Chu, Sudeep Sarma, Jeffrey J. Gray

**Affiliations:** Department of Chemical and Biomolecular Engineering, Johns Hopkins University, Baltimore, MD 21218, USA.

## Abstract

Diffusion models have shown promise in addressing the protein docking problem. Traditionally, these models are used solely for sampling docked poses, with a separate confidence model for ranking. We introduce DFMDock (Denoising Force Matching Dock), a diffusion model that unifies sampling and ranking within a single framework. DFMDock features two output heads: one for predicting forces and the other for predicting energies. The forces are trained using a denoising force matching objective, while the energy gradients are trained to align with the forces. This design enables our model to sample using the predicted forces and rank poses using the predicted energies, thereby eliminating the need for an additional confidence model. Our approach outperforms the previous diffusion model for protein docking, DiffDock-PP, with a sampling success rate of 44% compared to its 8%, and a Top- 1 ranking success rate of 16% compared to 0% on the Docking Benchmark 5.5 test set. In successful decoy cases, the DFMDock Energy forms a binding funnel similar to the physics-based Rosetta Energy, suggesting that DFMDock can capture the underlying energy landscape.

## 1 Introduction

### 1.1 Classical docking methods

Protein-protein docking predicts the structure of a protein complex from the structures of its individual unbound partners [1]. Classical methods involve two key components: (1) sampling algorithms, such as exhaustive global searches, local shape-matching, and Monte Carlo algorithms, generate possible docked structures, while (2) scoring functions evaluate these structures based on physical energy, structural compatibility, or empirical data [2]. An alternative approach is template-based docking, which leverages sequence similarity and evolutionary conservation [3]. However, these methods are time-consuming due to the extensive search and evaluation processes involved. Hence, this work aims to develop a fast and accurate deep-learning method for sampling and scoring protein complexes.

### 1.2 Related work

#### Co-folding models.

Co-folding models, which predict protein complex structure from sequences, have emerged as powerful tools for addressing protein docking. By leveraging large datasets of protein sequences [4] and structures [5], these models have shown remarkable accuracy in predicting protein complex structures. AlphaFold2 [6] and RoseTTAFold [7] marked a significant breakthrough in protein structure prediction. Originally developed for monomer structure prediction, extensions for multimers were released soon after, making these models the preferred approach for most protein complex predictions. However, challenges remain, such as the time-consuming multiple sequence alignment (MSA) searches and lower accuracy in predicting antibody-antigen interactions [8]. Recently, AlphaFold3 [9] introduced a diffusion module, replacing the previous structure module, and expanded its capabilities to predict interactions not only between proteins but also with DNA, RNA, and small molecules; but AlphaFold3 still fails in 40% of antibody-antigen cases.

#### Regression-based models.

Unlike co-folding models, regression-based models generally input the individual protein structures, either in 3D or as distance matrices, without relying on MSA. EquiDock [10] was the first model to apply equivariant neural networks for rigid protein docking. While its theoretical framework is robust, its success rate is lower compared to traditional and co-folding models. Following a similar approach, ElliDock [11] introduced elliptic-paraboloid interface representations but did not significantly improve performance. In contrast, GeoDock [12] and DockGPT [13] adopted architectures resembling AlphaFold2, using individual protein structures without MSA while allowing flexible backbones. Although they outperform EquiDock, these methods still underperform compared to co-folding approaches. The limitations of regression-based models are (1) they generate only a single prediction per model, and (2) they are less accurate for predicting protein interactions beyond the training data compared to co-folding models with MSAs.

#### Diffusion models.

Diffusion models [14–16] have been applied to protein docking [17]. Unlike regression-based objectives, DiffDock [18] reformulates docking as a generative process, training the model through denoising score matching [19] on the translation, rotation, and torsion spaces of small molecules. DiffDock-PP [20] adapts DiffDock for protein-protein docking and diffuses only along the translation and rotation spaces (rigid docking). DiffMaSIF [21] follows a similar framework but incorporates additional protein interface embeddings. LatentDock [22] applies diffusion in the latent space, first training a variational autoencoder [23] and then diffusing in the encoder’s output, akin to Stable Diffusion [24]. All these models comprise a sampling model, which generates diverse poses through reverse diffusion steps, and a confidence model, which ranks these poses based on their confidence scores.

#### Energy-based models.

Energy-based models [25] train neural networks to approximate the underlying energy function of the training data. DockGame [26] introduces a framework that trains energy functions either supervised by physics-based models or self-supervised through denoising score matching. EBMDock [27] employs statistical potential as its energy function and uses Langevin dynamics to sample docking poses. Arts *et al*. [28] developed diffusion model-based force fields for coarse-grained molecular dynamics by parameterizing the energy of an atomic system and training the gradient of the energy to match the denoising force. With this learned force field, they can both sample from the distribution and perform molecular dynamics simulations. Based on the theory that diffusion models learn the underlying training data distribution, which if well approximated can relate to the energy of the generated samples. DSMBind [29] adopts a similar framework for protein-protein interactions, demonstrating that the learned energy function correlates more strongly with binding energy than previous methods.

Building on these approaches, we propose DFMDock, a diffusion generative model for protein docking. To our knowledge, it is the first model to integrate sampling and ranking within a single framework, utilizing forces and energies learned from diffusion models. Our results show that DFMDock outperforms DiffDock-PP in both sampling and Top-1 ranking success rates. Furthermore, the learned energy function exhibits a binding funnel similar to the physics-based Rosetta energy function [30], suggesting that DFMDock can capture the underlying energy landscape for some protein-protein interactions.

## 2 Methods

### 2.1 Model

According to statistical physics, a docking pose x is a random state sampled from the Boltzmann distribution: $p(x)=\frac{e^{-E(x)/k_{B}T}}{Z}$, where E(x) is the energy, $k_{B}$ is the Boltzmann constant, T is the temperature, and $Z=\int_{\Omega }e^{-E(x)/k_{B}T}$ is the partition function. For rigid docking between a “receptor” protein and a “ligand” protein, the search space Ω spans all possible translations and rotations of the ligand, with the receptor fixed. The per-residue forces on the ligand are given by:

(1) $$f_{i}=-\nabla_{r_{i}}E(x)=\nabla_{r_{i}}\;\text{log}\;p(x),$$

where $r_{i}$ are the $C_{\alpha }$ coordinates of the ligand residues relative to their center. Our goal is to learn $f_{i}$ and E(x), using $f_{i}$ for sampling docking poses and E(x) for ranking.

We use an equivariant graph neural network (EGNN) [31] to predict the energy E and the forces $f_{i}$ on each residue:

(2) $$h^{L},x^{L}=\text{EGNN}\left(h^{0},x^{0},e_{\text{ij}}\right).$$

h represents the node embeddings, which for the input $h^{0}$ concatenate the amino acid sequence one-hot encoding with the ESM2 (650M) embeddings [32]. x is the set of $C_{\alpha }$ coordinates, and $e_{\text{ij}}$ is an edge embedding of trRosetta [33] geometry and relative positional encoding [34]. To balance short- and long-range interactions, we construct graphs of 20 nearest neighbor edges and 40 edges selected randomly using an inverse cubic distance weighting [35]. The predicted energy is computed as the average output of a multi-layer perceptron (MLP) $\phi_{E}$ that inputs the concatenated node representations of ligand i and receptor j, where the distance between them, $d_{\text{ij}}$, is within a cutoff distance d:

(3) $$E=\frac{1}{N}\sum_{\left(i,j\right):d_{\text{ij}}<d}\phi_{E}\left(h_{i}^{L}\left\|h_{j}^{L}\right.\right),$$

where N is the number of residue pairs within the cutoff distance. The predicted forces on each ligand residue are derived as the displacement vectors between the input coordinates and the updated coordinates:

(4) $$f_{i}=x_{i}^{L}-x_{i}^{0}.$$

The translational force for the ligand is obtained by averaging the per-residue forces: $F_{\text{translation}}=\frac{1}{n}\sum_{i=1}^{n}f_{i}$. In the Appendix, we show that the gradient of energy with respect to a rotation vector ω is given by $\nabla_{\omega }E=\frac{1}{n}\sum_{i=1}^{n}\left(r_{i}\times \nabla_{r_{i}}E\right)$. Thus, the rotational force for the ligand is: $F_{\text{rotation}}=-\nabla_{\omega }E=\frac{1}{n}\sum_{i=1}^{n}r_{i}\times f_{i}$.

To improve numerical stability [36], we normalize the translational and rotational forces and use two MLPs ϕ that input the unnormalized magnitude and timestep t, to learn the scaling factors:

(5) $${\overset{ˆ}{F}}_{\text{translation}}=\frac{F_{\text{translation}}}{\left\|F_{\text{translation}}\right\|}\cdot \phi_{\text{translation}}\left(\left\|F_{\text{translation}}\right\|,t\right),$$

(6) $${\overset{ˆ}{F}}_{\text{rotation}}=\frac{F_{\text{rotation}}}{\left\|F_{\text{rotation}}\right\|}\cdot \phi_{\text{rotation}}\left(\left\|F_{\text{rotation}}\right\|,t\right).$$

We train the model using denoising force matching for both translation and rotation:

(7) $$L_{\text{translation}}={\left\|{\overset{ˆ}{F}}_{\text{translation}}-\nabla_{\overset{ˆ}{x}}\text{log}\;p(\overset{ˆ}{x}|x)\right\|}^{2},$$

(8) $$L_{\text{rotation}}={\left\|{\overset{ˆ}{F}}_{\text{rotation}}-\nabla_{\overset{ˆ}{\omega }}\text{log}\;p(\overset{ˆ}{\omega }|\omega )\right\|}^{2},$$

where $p(\overset{ˆ}{x}|x)$ denotes the conditional probability distribution of the noised translation $\overset{ˆ}{x}$ given the true translation x, modeled as a Gaussian distribution in $R^{3}$, and $p(\overset{ˆ}{\omega }|\omega )$ represents the conditional probability distribution of the noised rotation $\overset{ˆ}{\omega }$ given the true rotation ω, modeled as an isotropic Gaussian distribution on SO(3) [37–39].

To train the energy function for ranking, the energy conservation loss [40] is calculated as the mean squared error between the predicted forces and the negative gradient of the predicted energy with respect to the coordinates:

(9) $$L_{\text{conservation}}={\left\|-\nabla_{r_{i}}E(x)-f_{i}\right\|}^{2}.$$

To ensure that the global energy minimum aligns with the ground truth [41, 42], the energy contrastive loss is defined as:

(10) $$L_{\text{contrastive}}=-\text{log}\left(\frac{e^{-E_{\text{gt}}}}{e^{-E_{\text{gt}}}+e^{-E_{\text{noised}}}}\right),$$

where $E_{\text{gt}}$ and $E_{\text{noised}}$ are the energies of the ground truth and noised structures. The final loss function combines these components:

(11) $$L=L_{\text{translation}}+L_{\text{rotation}}+L_{\text{conservation}}+L_{\text{contrastive}}.$$

### 2.2 Data

We trained our model on DIPS-hetero, a subset of DIPS [43, 44] with approximately 11k heterodimers. For testing, we evaluated it on 25 targets, as selected in the EquiDock report, from the Docking Benchmark 5.5 (DB5.5) [45], a widely used dataset for assessing docking performance.

## 3 Results and Discussion

### 3.1 DFMDock achieves higher success rate than DiffDock-PP

**Figure 2:**
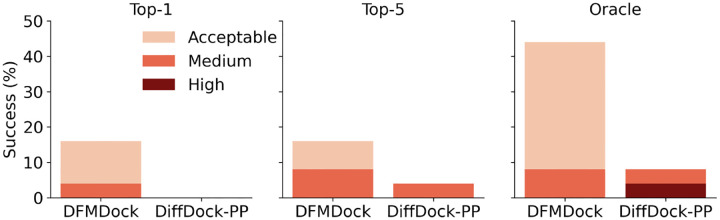
Success rates of DFMDock and DiffDock-PP in Top-1, Top-5, and Oracle settings, categorized by Acceptable (DockQ > 0.23), Medium (DockQ > 0.49), and High (DockQ > 0.80) accuracy ratings [46].

We compared DFMDock to DiffDock-PP (trained on DIPS) on the DB5.5 test set. Both models generated 120 samples per target, each initialized from different starting positions, using 40 diffusion steps. DFMDock samples were ranked using the model’s energy function (DFMDock Energy) and DiffDock-PP samples were ranked using it’s confidence model. As shown in Figure 2, DFMDock consistently outperforms DiffDock-PP across all settings, with the largest margin observed in the Oracle setting. (Here Oracle refers to the highest DockQ among all samples per target). While DiffDock-PP achieved state-of-the-art success on the DIPS test set, its performance on DB5.5 dropped significantly (8%), likely due to data leakage between the DIPS training and test sets [12, 47]. In contrast, DFMDock demonstrates better generalization to protein-protein interactions in DB5.5.

### 3.2 DFMDock learns physics-like energy

**Figure 3:**
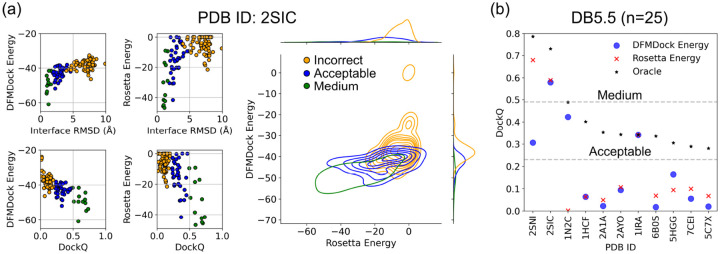
Comparison of ranking methods between DFMDock and Rosetta. (a) Binding funnels (Interface RMSD and DockQ) and contour plot (DFMDock Energy vs Rosetta Energy) for a target from the DB5.5 test set (PDB ID: 2SIC), colored by DockQ ranges: Incorrect (orange), Acceptable (blue), and Medium (green). (b) Comparison of Top-1 ranked DockQ scores for different PDB IDs: DFMDock Energy (blue circles), Rosetta Energy (red crosses), and Oracle (black stars). The dashed line at DockQ=0.23 and DockQ=0.49 indicates the Acceptable and Medium threshold. Only successful decoy sampling cases from DB5.5 (11 out of 25) are shown.

To evaluate our model’s energy function, we plotted both the DFMDock Energy and the Rosetta Energy vs the Interface RMSD and DockQ (two measures of docking accuracy) for the 120 DFMDock-generated samples per target. Figure 3a shows similar binding funnels under both scoring methods for a sample target (PDB ID: 2SIC), suggesting that DFMDock captures the physical energy landscape of protein docking. This funnel-like behavior indicates the model can distinguish between nearnative and non-native docking poses, making it valuable for ranking docking decoys. The contour plot of Rosetta Energy vs DFMDock Energy shows that DFMDock ranks medium and acceptable quality predictions higher than incorrect predictions. Figure 3b shows that DFMDock energy slightly outperforms Rosetta in identifying acceptable quality poses (4 vs 3 targets) but underperforms in discriminating medium quality structures (1 vs 2 targets). In 7 out of 11 cases where DFMDock succeeded in sampling, both scoring methods failed to rank the poses correctly, suggesting that the sampled poses may be of lower quality or contain steric clashes, thus requiring further development of the model.

**Figure 4:**
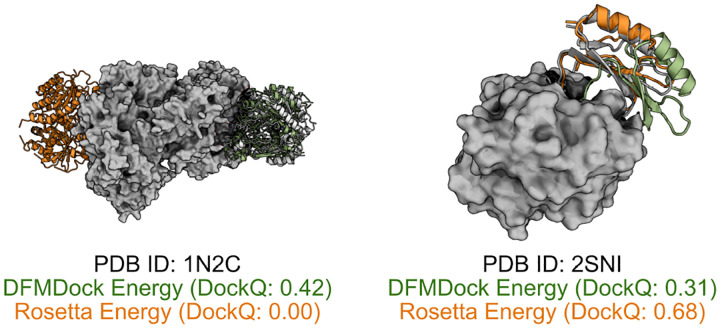
Top-1 predictions ranked by DFMDock Energy (green) and Rosetta Energy (orange) aligned to the ground truth structures (grey).

Figure 4 shows a structural comparison between DFMDock and Rosetta’s top-ranked predictions for two samples. For PDB ID 1N2C, DFMDock identifies an acceptable quality pose (DockQ=0.42), while Rosetta identifies an incorrect pose (DockQ=0.00). However, for 2SNI, DFMDock fails to identify medium quality structures as effectively as Rosetta energy, suggesting that DFMDock’s energy function is less accurate in this case. Incorporating all-atom details into DFMDock’s energy function could help address this issue, enhancing its ability to distinguish between acceptable and medium-quality docking poses and improving its overall reliability across diverse protein-protein interactions.

## 4 Conclusion

DFMDock is a generative diffusion model that integrates sampling and ranking for protein docking. DFMDock outperforms DiffDock-PP on the DB5.5 test set and generates binding funnels comparable to those from the Rosetta interface score, highlighting its ability to mimic physical interactions. However, in many cases where DFMDock succeeded in sampling, it failed to rank poses accurately, indicating the need for further development. Additionally, its accuracy in identifying medium-quality structures can be further optimized. Currently, the model is trained on a limited dataset, which may constrain its generalization. With the availability of larger and more comprehensive datasets [48], future work will incorporate all-atom details to enhance DFMDock’s precision and reliability across a broader range of protein interactions.

## Figures and Tables

**Figure 1:**
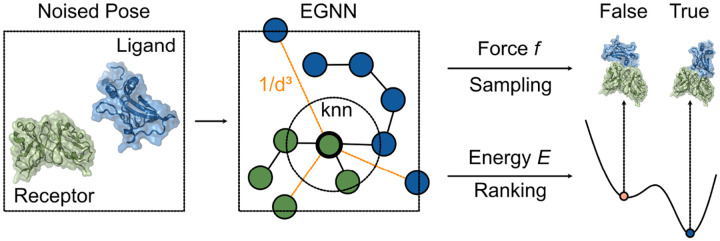
DFMDock model overview.

## Appendix

### Gradient of energy E with respect to a rotation vector ω

Consider a rigid body with N points labeled $r_{1},r_{2},\ldots ,r_{N}$, where each $r_{i}=x_{i}-c$ represents the position of point i relative to the center of mass c. When the rigid body undergoes a small rotation dω, where ω is a rotation vector, the displacement $dr_{i}$ of each point $r_{i}$ is given by:

$$dr_{i}=d\omega \times r_{i}$$

The energy E of the system depends on the positions $r_{i}$. The change in energy dE due to small displacements $dr_{i}$ of the points is:

$$dE=\sum_{i=1}^{N}\nabla_{r_{i}}E\cdot dr_{i}$$

where $\nabla_{r_{i}}E$ is the gradient of the energy with respect to the position of point $r_{i}$. (In this work we assume the direct energy dependence on rotation angle of each residue is small.)

Substituting the expression for the displacement $dr_{i}$, we get:

$$dE=\sum_{i=1}^{N}\nabla_{r_{i}}E\cdot \left(d\omega \times r_{i}\right)$$

Using the scalar triple product identity:

a⋅(b×c)=b⋅(c×a)

we can simplify the expression for dE:

$$dE=\sum_{i=1}^{N}d\omega \cdot \left(r_{i}\times \nabla_{r_{i}}E\right)$$

Thus, the gradient of energy with respect to the rotation vector ω is:

$$\nabla_{\omega }E=\sum_{i=1}^{N}\left(r_{i}\times \nabla_{r_{i}}E\right)$$

where $r_{i}$ is the vector from the center of mass to the point $x_{i}$, and $\nabla_{r_{i}}E$ is the gradient of the energy with respect to the position of point $r_{i}$.

If we have a finite number of points N, we can normalize the sum by the number of points to get an average gradient:

$$\nabla_{\omega }E=\frac{1}{N}\sum_{i=1}^{N}\left(r_{i}\times \nabla_{r_{i}}E\right)$$

This provides an averaged estimate of the gradient of energy with respect to the rotation vector ω when dealing with a finite set of points on the rigid body.
